# Genetic therapies for movement disorders - current status

**DOI:** 10.1007/s00415-025-12940-5

**Published:** 2025-02-22

**Authors:** J. Sartorelli, J. Ng, A. A. Rahim, S. N. Waddington, M. A. Kurian

**Affiliations:** 1 Developmental Neurosciences, Zayed Centre for Research Into Rare Disease in Children, UCL Great Ormond Street Institute of Child Health, 20 Guilford Street, London, WC1N 1DZ UK; 2 Unit of Muscular and Neurodegenerative Diseases, Bambino Gesù Children’s Hospital IRCCS, Rome, Italy; 3 Genetic Therapy Accelerator Centre, UCL Queen Square Institute of Neurology, London, UK; 4 UCL School of Pharmacy, University College London, London, UK; 5 Gene Transfer Technology Group, Institute for Women’s Health, University College London, London, UK; 6 Wits/SAMRC Antiviral Gene Therapy Research Unit, Faculty of Health Sciences, University of the Witwatersrand, Johannesburg, South Africa; 7 Department of Neurology, Great Ormond Street Hospital, London, UK

**Keywords:** Genetic therapy, Movement disorder, Gene, Viral vector, Brain

## Abstract

Movement disorders are a group of heterogeneous neurological conditions associated with alterations of tone, posture and voluntary movement. They may either occur in isolation or as part of a multisystemic condition. More recently, the advent of next generation sequencing technologies has facilitated better understanding of the underlying causative genes and molecular pathways, thereby identifying targets for genetic therapy. In this review, we summarize the advances in genetic therapy approaches for both hyperkinetic and hypokinetic movement disorders, including Parkinson’s Disease, Huntington’s Disease and rarer monogenic conditions of childhood onset. While there have been significant advances in the field, multiple challenges remain, related to safety, toxicity, efficacy and brain biodistribution, which will need to be addressed by the next generation of genetic therapies.

## Introduction

Movement disorders comprise a heterogeneous group of conditions characterized by abnormalities in tone, posture and voluntary movement. Movement disorders may be hyperkinetic (e.g., dystonia, chorea, myoclonus) or hypokinetic (e.g., parkinsonism) and may occur in isolation or as part of a more complex neurologic condition, often with multiple phenomenologies and systemic involvement [1]. They may be genetic (monogenic, polygenic risk or susceptibility), acquired (infectious, post-infectious, environmental) or associated with functional and neuropsychiatric disease [2–4]. Movement disorders arise from disruption of networks within the basal ganglia, thalamic, cortical and cerebellar circuitry [5, 6].

Over the last decade, the advent of next generation sequencing technologies has allowed better understanding of many movement disorders, thereby driving an era of precision medicine, targeted at treating the underlying etiology. For some conditions, genetic therapy is a highly promising strategy, where gene supplementation, modification or editing is achieved by transfer of a gene or genetic material to the desired brain region using viral and non-viral vector delivery systems.

Within the clinical setting, a broad range of viral vectors have been used, most commonly lentiviral and AAV vectors. Lentiviruses are a genus of retroviruses, which possess an RNA genome that undergoes reverse transcription before integrating into the host genome; they have been used for both targeted brain delivery and ex vivo approaches [7, 8]. Wildtype adeno-associated virus (AAV) are parvoviruses of the *Dependoparvovirus* genus and carry a single-stranded DNA genome which integrates into the host genome. In contrast, recombinant AAV vectors lack the integration machinery and instead they remain predominantly episomal. AAV vectors have also been used in the clinic, with a broad range of capsid variations, natural and synthetic, with different tissue tropism [9–11]. Over the last decade, RNA therapies and CRISPR-based gene editing strategies are also emergent approaches.

**Fig. 1:**
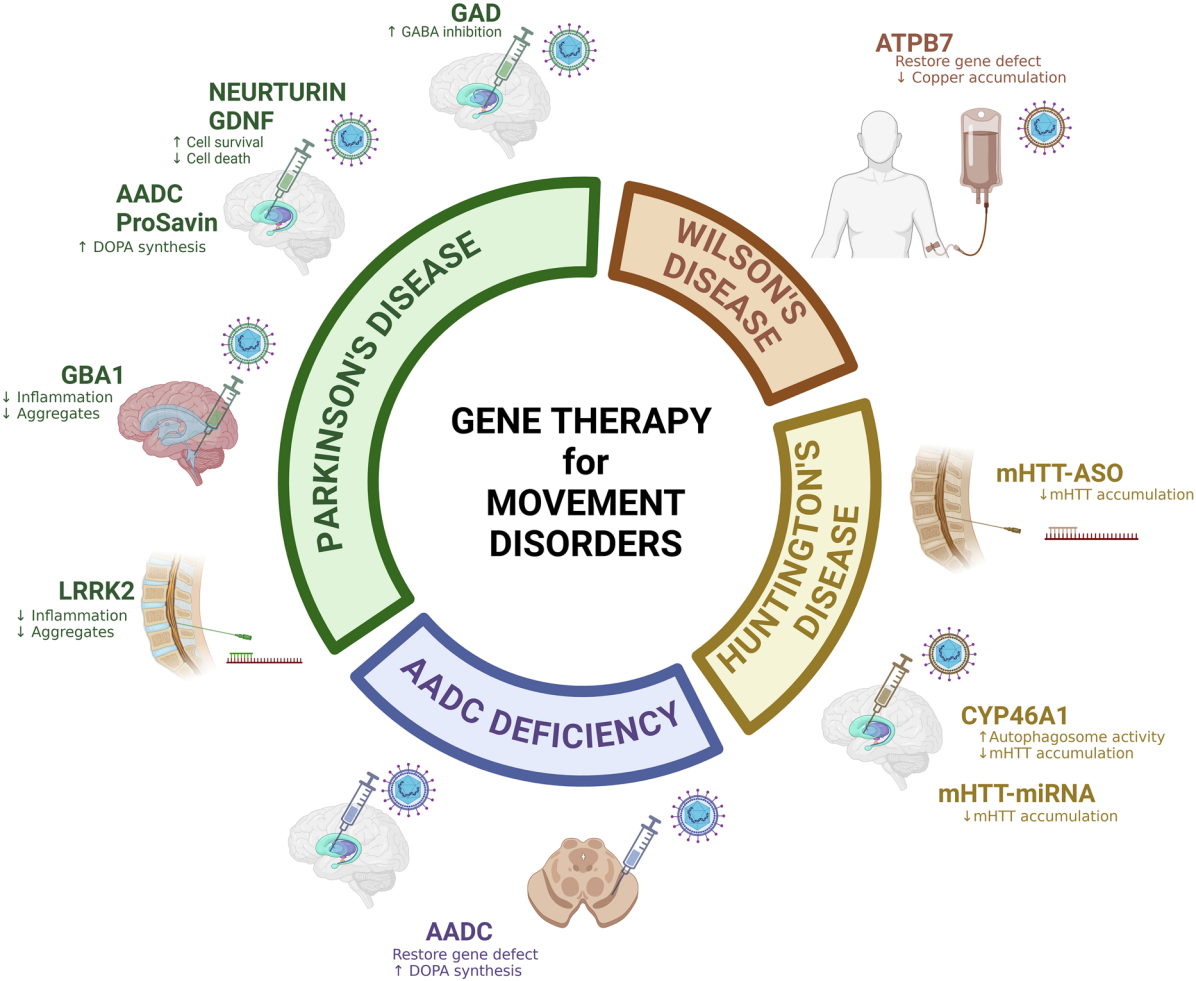
Genetic strategies for the treatment of movement disorders: Genetic strategies for common complex and rare monogenic movement disorders include viral-vector mediated DNA-based gene therapy and RNA-based therapies (Created in https://BioRender.com)

Our review aims to highlight how genetic therapies (DNA and RNA strategies) are now being explored for movement disorders, using specific common and rare disease examples (Fig. 1).

## Gene therapy for Parkinson’s Disease

Parkinson's Disease (PD) is a neurodegenerative disease characterized by progressive loss of dopaminergic neurons in the midbrain [12]. The major motor manifestations of PD include tremor, rigidity, akinesia, gait, and postural disturbance, but non-motor manifestations such as neuropsychiatric symptoms, sleep disorders and autonomic dysfunction can also present, and may even precede motor symptoms [13]. The current mainstay of treatment is to increase available dopamine through L-dopa supplementation. However, with disease progression over time, on–off phenomena, waning drug efficacy and intolerable side-effects necessitate other therapeutic approaches such as Deep Brain Stimulation (DBS) as palliative measures [14]. The development of disease-modifying therapies constitutes a research priority for PD.

### GAD gene therapy

The first trial of gene therapy for PD proposed to deliver rAAV vectors expressing the two isoforms of the enzyme glutamic acid decarboxylase (*GAD-65* and *GAD-67*), which synthesize the major inhibitory neurotransmitter, gamma aminobutyric acid (GABA) targeting the subthalamic nucleus (STN) [15]. The STN is disinhibited in PD, leading to pathologic excitation of the internal segment of the globus pallidus (GPi) and substantia nigra pars reticulata (SNpr). The aim of this therapy was to convert excitatory STN projections to inhibitory, to slow down the degeneration of dopaminergic neurons as shown in preclinical studies [15–17].

The first open-label phase 1 trial (NCT00195143) assessed unilateral subthalamic AAV-GAD injection in 12 patients with PD. There were no adverse events related to gene therapy, with significant improvements in motor capacity assessed through the Unified Parkinson Disease Rating Scale (UPDRS) scores, predominantly on the side of the body that was contralateral to surgery from 3 months up to 12 months after gene therapy [18]. Furthermore, functional studies with fluorodeoxyglucose (FDG) positron emission tomography (PET) undertaken before and after gene therapy showed a post-operative reduction of abnormally elevated metabolic activity in networks associated with motor and cognitive functioning, in particular, the motor side after therapy, persisting for 1 year. These network changes also correlated with improved clinical disability ratings [19]. Subsequently, a double-blind, randomized controlled phase 2 trial was undertaken (NCT00643890): patients were randomized to AAV2-GAD therapy or sham surgery. At the 6-month endpoint, UPDRS score decreased in both treated and sham group, with a significantly greater improvement from baseline in AAV2-GAD group. No serious adverse events (SAEs) were attributed to treatment or the surgical procedure. In patients excluded from the analysis because of poor catheter tip location or infusion pump failure, UPDRS motor scores either increased or did not change in four of five patients while one had a 21% decrease in score. Inclusion of all these patients in an intention-to-treat analysis showed no significant difference between groups [20]. At 12 months there was persistent improvement in UPDRS motor scores in the AAV2-GAD group compared with the sham group, and a significant decline of levodopa-induced dyskinesias. Analysis of FDG PET images revealed significant metabolic decline in the thalamus, striatum, and prefrontal, anterior cingulate, and orbitofrontal cortices in the AAV2-GAD group, with a correlation between baseline metabolism in the prefrontal cortex (PFC) and changes in motor UPDRS scores: the higher the baseline PFC metabolism, the better the clinical outcome [21]. Patients who received GAD gene therapy developed a unique treatment-dependent polysynaptic brain circuit termed as the GAD-related pattern (GADRP), which appeared to correlate with clinical improvement, reflecting the formation of new polysynaptic functional pathways linking the STN to the premotor region [22]. Results of a five-year follow-up study (NCT01301573) of this cohort are still to be published. A new AAV-GAD therapy phase 1–2 study (NCT05603312) has been completed in early September 2024, with promising results as reported by the sponsor in advance of complete data report [23]; a long-term follow-up study (NCT05894343) is currently in enrollment.

### Neurturin and GDNF

Neurotrophic factors have been shown to protect degenerating neurons in vitro from further damage. *GDNF* is effective in enhancing the function of nigrostriatal dopaminergic neurons in preclinical models [24]. Neurturin (*NRTN*), a natural structural and functional analog of *GDNF*, also promotes survival of dopaminergic neurons both in rodents and primates [25, 26]. A first Phase 1 open-label study (NCT00252850) evaluated the feasibility of bilateral, stereotactic, intraputaminal injection of AAV2-neurturin (CERE-120) at two different concentrations. The procedure was well tolerated in participants, with no clinically significant adverse events and partial improvement in some secondary measures of motor function at 1 year (UPDRS off-medication motor subscore improvement and an increment in time without troublesome dyskinesia in patients reports). (18)F-levodopa-uptake PET did not change after treatment with either dose of CERE-120 [27]. A subsequent phase 2 randomized, multicenter, double-blind controlled trial (NCT00400634) was undertaken, but there was no significant difference in the primary endpoint markers of efficacy. Transient SAEs occurred almost in a third of treated patients, related to the surgical procedure, and included confusion, seizure, hemorrhage, cerebral edema, and caudate nucleus infarct (all in one patient), transient cognitive change (in one patient), and urinary retention (in two patients). Two deaths occurred in the treatment group, unrelated to CERE-120. One treated patient had a glioblastoma that was present before treatment. Analysis of post-mortem brain tissue from treated patients with NRTN-immunostaining showed striatal coverage comparable to that in the primate model, but significantly lower expression in the substantia nigra pars compacta (SNc), suggesting that retrograde transport did not occur [28].

As a result, a new Phase 1 study targeting CERE-120 to both the putamen and the substantia nigra was undertaken. Two-year safety data showed that the procedure was well-tolerated, with no SAEs. All transient adverse events were deemed related to stereotactic brain surgery, including incision site pain, dyskinesia, headache, and abnormal dreams. The motor-off UPDRS and off-time on self-reported home diary records showed a slight improvement over time which persisted up to 24 months [29]. A Phase 2 double-blind trial was then implemented but there was no difference between the treated and placebo group in either the primary endpoint (motor-off UPDRS) or in most secondary endpoints [30]. A long-term follow-up study up to 5 years post-treatment showed no clinically relevant changes in examination, imaging, or laboratory studies and only a stable or modest improvement in motor status [31]. Post-mortem brain analysis of two patients with advanced Parkinson Disease 8 and 10 years following CERE-120 gene therapy evidenced limited NRTN expression [32].

GDNF gene therapy was also investigated and shown to be more potent than NRTN in preclinical studies, though it lacked efficacy when infused into humans as a protein via intracerebroventricular infusion [33]. A first gene therapy trial evaluated MRI-guided putaminal infusion of AAV2-GDNF. UPDRS scores remained stable and almost all patients had increased (18)FDOPA-PET uptake up to 18 months post-infusion [34]. Advances in imaging allowed MRI-guided infusions to be undertaken (gadoteridol co-infusion) and real-time intraoperative MRI to confirm infusion cannula placement, anatomic quantification of volumetric perfusion within the putamen, and direct visualization of off-target leakage or cannula reflux, permitting infusion rate and cannula adjustments. Serial post-treatment MRI demonstrated no evidence of cerebral parenchymal toxicity in the corresponding regions of AAV2-GDNF and gadoteridol co-infusion nor in surrounding regions over long-term follow-up [35]. The true efficacy of *GDNF* gene therapy efficacy remains yet to be determined and a Phase 1 clinical trial is still ongoing (NCT04167540), with early reports of good tolerability for intraputaminal injections, good coverage and long-term follow-up ongoing. Recruitment for a Phase 2 study is ongoing (NCT06285643).

#### AADC

The *DDC* gene encodes aromatic l-amino acid decarboxylase (AADC) which catalyzes the decarboxylation of L-3,4-dihydroxyphenylalanine (L-DOPA) to dopamine and L-5-hydroxytryptophan to serotonin [36]. Due to its key role in dopamine synthesis, AADC gene therapy has been investigated in primate models of PD, targeting striatal cells (aiming to replenish dopamine in a depleted system from SNc degeneration), with concomitant administration of lower doses of L-dopa [37]. These studies led to phase I clinical trials assessing bilateral putaminal infusion of different doses of AAV2-AADC vector. PET scans using the AADC tracer, 6-[18F]fluoro-l-m-tyrosine (FMT) were used for assessment of enzyme activity. All studies showed an increase in FMT activity, proportional to AAV2-AADC dosage, which persisted up to 96 weeks post-infusion. Preliminary clinical data showed increased on-time and reduced off-time without increased “on” time dyskinesia. The procedure was well tolerated except for 3 intracranial hemorrhages [38–40]. Long-term follow-up revealed persistent FMT-PET signal increment over 4 years. The UPDRS scale performed 12 h after last dopaminergic medication improved in all patients in the first 12 months, but displayed a slow deterioration in subsequent years [41].

Imaging analysis highlighted the accuracy of MRI-guided infusion for drug-volume optimization: researchers found increased PET uptake at the sites of T2 hyperintensity which also correlated to immunohistochemistry images in brains of nonhuman primates [42]. A subsequent phase 1 trial with 3 incremental doses of VY-AADC01 (AAV2-hAADC) then evaluated the efficacy of MRI-guided infusion in bilateral putamen, showing regional coverage proportional to drug dosage (up to 42%) and an increase in corresponding enzyme activity assessed by FMT-PET (up to 79%), leading to a decrease in antiparkinsonian medications and drug-related dyskinesia, which lasted up to 1 year post-treatment [43]. A study with intravenous levodopa administration showed that VY-AADC01 efficacy was greater with longer lasting effect on UPDRS scores with lower doses of levodopa supplementation [44]. 36 months follow-up showed a good safety profile, with no SAEs attributed to VY-AADC01. Requirements for PD medications were reduced by 21–30% in the 2 highest dose cohorts and standard measures of motor function (PD diary, UPDRS III off and on-medication scores), global impressions of improvement (Clinical Global Impression of Improvement, Patient Global Impression of Improvement), and quality of life (39-item Parkinson Disease Questionnaire) were stable or improved when compared to baseline at all time points up to 36 months across all cohorts [45]. Currently a Phase 1 study is ongoing (NCT03562494) in PD patients with motor fluctuations, with an expected completion date in November 2024.

### ProSavin

A different combined approach to restore dopamine production was investigated using a lentiviral vector carrying the rate-limiting dopamine synthesis enzyme, tyrosine hydroxylase, with AADC, and GTP cyclohydrolase 1, shown sufficient for dopamine production in nondopaminergic neurons in preclinical studies [8]. This vector (ProSavin) was injected bilaterally into the putamen of PD patients in a Phase 1/2 open-label trial with three incremental doses and, despite mild and moderate adverse events (mainly increased on-medication dyskinesias and on–off phenomena), no SAEs related to the study drug or surgical procedure were reported. A significant improvement in mean UPDRS part III motor scores off medication was recorded in all patients at 6 and 12 months, when compared with baseline [46]. In the long-term follow-up, dyskinesias resolved with a reduction of oral dopaminergic medications. On–off phenomena persisted especially in the first year. Motor score improvement was still seen at 2 years and persisted at 4 years in almost half of treated patients [47]. The authors concluded that “higher levels of dopamine replacement may be required to maximize the clinical benefit” thus proceeding to develop an optimized construct, OXB-102, to yield higher dopamine production [48–50].

### Future perspectives

Several new gene therapy approaches are in the first phases of clinical development.

*GBA1* encodes glucosylceramidase (GCase), a lysosomal enzyme with an essential role in glycolipid breakdown and protein homeostasis. *GBA1* mutations lead to Gaucher disease and confer higher risk for PD, due to a putative role in alpha-synuclein accumulation [51]. Preclinical studies in multiple models have shown increasing GCase levels and enzyme activity after delivery of a *GBA1* vector, which was also seen to reduce inflammation and accumulation of aggregates. [52–54]. A subsequent Phase 1/2 trial then evaluated the safety of PR001, an AAV9-GBA1 gene therapy in patients with PD harboring at least one *GBA1* mutation, delivered by intra-cisterna magna injection [55]. In August 2020, however, a patient with PD, approximately three months following PR001 administration, experienced SAEs presumably associated with an immune-mediated response to the AAV9 viral vector, which required additional immunosuppressive treatment. The clinical protocol was therefore modified to optimize the immunosuppression regime, and a modified open-label trial is actively recruiting (NCT04127578) [56].

Missense mutations in the leucine-rich repeat kinase 2 (*LRRK2*) gene cause monogenic PD. Notably, *LRKK2* knockdown has been shown to protect dopaminergic neurons from neurodegeneration in animal models, most likely associated to reduced neuroinflammation and alpha-synuclein accumulation [57]. In preclinical studies, *LRRK2* inhibition through an antisense oligonucleotide (ASO) approach leads to reduction in both LRRK2 protein and alpha-synuclein inclusions and it has also been translated in a Phase 1 clinical trial evaluating increasing ASO doses administered via intrathecal injections in patients with PD which has been completed in August 2024 (NCT03976349).

## AADC deficiency

After the promising results of *hAADC* gene therapy in PD patients, a similar approach has been trialed in patients with aromatic L-amino acid decarboxylase deficiency (AADCD; OMIM #608643), a rare autosomal recessive neurotransmitter disorder that leads to a severe combined deficiency of serotonin, dopamine, norepinephrine and epinephrine. Symptom onset typically occurs during the first few months of life, with most patients presenting with a severe phenotype characterized by early-onset hypotonia, oculogyric crises, ptosis, dystonia, hypokinesia, delayed development and autonomic dysfunction [58–61].

Standard therapy with dopaminergic agents and symptomatic therapies provides only modest benefit, and as such, AADCD is associated with significant morbidity and a high risk of childhood mortality. The first AADCD patients to be treated with gene therapy received viral vector–mediated gene transfer of the human *AADC* gene bilaterally into the putamen; four patients, aged 4 to 6 years of age were treated through a compassionate use program. In a 12 month follow-up study, all patients showed improvements in motor performance: one patient was able to stand 16 months after gene therapy, and the other three patients achieved supported sitting 6 to 15 months post-gene therapy. AADC enzyme activity was confirmed through PET and CSF analysis, which showed increased dopamine and (unexpectedly) serotonin levels. Adverse effects included transient generalized dyskinesia which tended to improve after several months, and episodes of apnea in one patient, which again diminished 10 months after therapy [62].

After these promising results, two Phase 1/2 trials were conducted, enrolling patients with a confirmed AADCD diagnosis, age 2 to 19 years. Motor function improved in all patients up to 2 years after gene therapy and AADC activity was confirmed by an increase in CSF homovanillic acid levels and increased tracer uptake in the putamen on FMT-PET. Patients with an early-onset severe phenotype were able to sit with support or in some cases walk with a walker post-gene therapy. One patient with a more moderate phenotype was able to run and ride a bicycle and showed an improvement in cognitive abilities. Dystonia improved and oculogyric crises were markedly reduced in all patients, especially in the younger patients. Transient post-gene therapy dyskinesia was observed in all patients but resolved within months. Other frequently reported adverse events included pyrexia which was also transient [63, 64]. Long-term follow-up showed improvements in motor and cognitive function within 12 months of gene therapy, which were sustained for more than 5 years in patients of all ages. Patient symptoms such as mood, sweating, temperature control and weight gain, as well as care giver quality of life also improved post-gene therapy, with reported better outcome in younger participants [65, 66].

As a result of these clinical trials, Eladocagene exuparvovec (Upstaza) received authorization both from the EMA and MHRA in 2022. In the UK and Europe, it is licensed for the treatment of patients aged above 18 months with a clinical, molecular, and genetically confirmed diagnosis of severe AADCD, through bilateral stereotactic intraputaminal infusion [67]. Furthermore, the FDA have also recently (November 2024) approved Eladocagene exuparvovec-tneq (Kebilidi) for the treatment of adult and pediatric patients with aromatic L-amino acid decarboxylase (AADC) deficiency.

An alternative approach for AADCD gene therapy has also been investigated using AAV2-hAADC to target the SNc and the ventral tegmental area (VTA) of the midbrain. The rationale for this approach is based on the structural integrity of midbrain dopaminergic neurons and their axonal projections in children with AADC deficiency (where neurodegeneration is not reported) as well as the potential for anterograde transport to neuroanatomically relevant regions like the striatum. Pearson and colleagues completed a Phase 1/2 study with MR-guided delivery of AAV2-hAADC to the midbrain in children aged 4 to 9 years. The procedure was safe, well-tolerated and achieved target coverage of 98% and 70% of the SNc and VTA, respectively. Dopamine production was increased in all subjects as evident on CSF neurotransmitter analysis and F-DOPA tracer uptake was also enhanced within the midbrain and the striatum. Oculogyric crisis resolved completely in 6 of the 7 subjects by 3 months post-surgery. After 12 months, 6 of 7 subjects gained full head control and 4 of 7 could sit independently. At 18 months, 2 subjects could walk with 2-handed support [68].

Finally, another different approach is being explored for AADCD. A Phase 1 trial evaluating putaminal delivery of AAV9-AADC (VGN-R09b) has recently commenced in China (NCT05765981). The study is focused on children with severe classical AADCD, above 2 years and under 8 years of age harboring at least one common founder splice site variant (IVS6 + 4A > T). The study aims to investigate motor development 12 months post-therapy.

## Huntington's disease

Huntington’s disease (HD) is an inherited autosomal dominant neurodegenerative condition caused by an expanded CAG trinucleotide repeat in the *HTT* gene. Triplet expansion in mutant huntingtin (mHTT) alters protein transcription and subsequent modifications interferes both with immune and mitochondrial function. This leads to neurodegeneration, resulting in devastating cognitive, psychologic, and motor deterioration [69]. Although clinical symptoms have been traditionally attributed to the progressive loss of medium-spiny GABAergic neurons of the striatum, HD is increasingly recognized as a disease of the whole brain and body involving different systems including the heart, skeletal muscle, thyroid, liver, and digestive tract [70, 71]. To date, the only treatment is symptomatic, and this severe condition is life-limiting, causing significant morbidity.

The main genetic approaches aimed at reducing the amount of mHTT involve targeting mRNA through RNA interference, microRNA, ASOs or small molecules in preclinical models. AMT-130, an AAV-microRNA has been shown to reduce both mHTT protein and mRNA levels in rat and minipig models, with widespread gene expression using an AAV5 vector [72, 73]. A proof-of concept study was started in the US evaluating safety and efficacy of intrastriatal AMT-130 administration in adults with early manifest HD (NCT04120493). The sponsor reported promising results from interim analysis while follow-up is still ongoing and now, a second study is actively recruiting in Europe to expand the number of patients involved with 5 years duration (NCT05243017). A second gene therapy named VY-HTT01, an AAV1-miRNA to be injected intraparenchymally was developed to clinical trial stage (NCT04885114). However, the study was withdrawn after the decision to switch to a new AAV vector for intravenous administration [74].

The first ASO applied in clinical trials was IONIS-HTT_Rx_, an oligonucleotide able to bind to its cognate mRNA, triggering RNase H1-mediated degradation of mHHT mRNA as shown in preclinical studies [75, 76]. A phase 1-2a trial and its subsequent open-label extension study showed a good safety profile, with low-grade adverse effects mainly linked to the lumbar puncture procedure, and a dose-dependent efficacy in lowering mHTT CSF concentration with an 8-week dosing schedule [77, 78]. After company acquisition, the same compound named RO7234292 or Tominersen was trialed in a Phase 3 study involving adults with manifest HD randomized to one of two different dose schedules or placebo with composite Unified Huntington’s Disease Rating Scale (cUHDRS) or Total Functional Capacity (TFC) scale at week 101 compared to baseline, as the primary endpoint. The trial was stopped after an interim analysis performed at week 69, evidencing worse or similar cUHDRS and TFC scores compared with placebo, with the same reduction in CSF mHTT evidenced in the pivotal study [79]. Post-hoc analysis and lower mHTT accumulation showed a possible benefit in younger HD patients, that is under investigation in a Phase 2 dose-finding trial which is now recruiting individuals with prodromal or early manifesting HD (NCT05686551).

A second oligonucleotide, WVE-003, has been developed to selectively target mHTT through a single nucleotide polymorphism (SNP3) specific for the mutant allele and it has been evaluated in a Phase 1/2 study, completed in May 2024. Each patient enrolled has been evaluated for presence of SNP3 in the expanded CAG allele, and based on available studies, up to 40% of HD patients would be eligible for this therapy [80, 81]. Early data report a good safety profile, a mean mHTT reduction of 44–46% with wild type HTT preservation and with a promising slowing of caudate atrophy [82]. A similar approach was also tried with different oligonucleotides, for which clinical trials were terminated due to lack of efficacy (NCT03225846, NCT04617847, NCT04617860) [78].

VO659, an ASO able to target CAG repeats in different mRNAs, is under evaluation in a Phase 1/2 study involving patients affected by HD and other neurodegenerative conditions (spinocerebellar ataxia type 1 and 3, NCT05822908).

Finally, a therapeutic approach through supplementation of the gene encoding cholesterol-24-hydroxylase (*CYP46A1*) has been investigated for HD, based on the rationale that this gene codes for a neuron-specific enzyme which has a role in brain cholesterol synthesis and metabolism, and is deficient in patients with HD. The AAV therapy AB-1001 led to favored autophagosome activity, reducing mutant Huntingtin protein aggregation and improving neuronal survival in a preclinical model [83]. This has now progressed to a Phase 1/2 clinical trial where the viral vector is targeted to the striatal nuclei (NCT05541627) [84].

## Wilson’s disease

Wilson’s disease (WD) is an inherited disorder caused by pathologic copper accumulation, leading to a wide range of symptoms, mainly linked to liver (hepatitis, jaundice) and brain disease (progressive tremor, dystonia, parkinsonism, dysarthria, gait and posture disturbance, drooling and dysphagia) [85, 86]. It is caused by biallelic loss-of-function mutations in *ATP7B*, a copper transporter for biliary excretion and mediating caeruloplasmin synthesis [87]. Systemic gene therapy has been successfully investigated for Wilson disease in a preclinical model [88]. However, the large gene size makes integration into an AAV vector challenging and so different ATPB7 isoforms have been studied [89]. Two clinical trials are now ongoing. The first one is a Phase 1/2/3 clinical trial evaluating the safety and efficacy of UX701, an AAV9 gene therapy designed to deliver a modified form of the *ATP7B* gene through a single IV infusion (NCT04884815) [90]. Early data report possible improvements in copper metabolism, with 6 out of 15 patients with Stage 1 WD (initial liver copper accumulation) who discontinued standard-of-care treatment with chelators and/or zinc therapy [91]. The second study is a Phase 1/2 trial evaluating VTX-801 (NCT04537377), an AAV vector encoding a mini version of human *ATP7B* which has been shown to restore copper metabolism in a murine model of WD [92]. Interim data reported adverse reactions linked to the AAV vector, like transient liver enzyme elevation but also a promising increment of ceruloplasmin ferroxidase activity together with normalization of liver fibrosis and copper accumulation at liver biopsy [93].

Two further studies have been registered in China: the first one (NCT06663878) registered and not yet recruiting, regarding an unspecified non-replicating gene transfer vector, named MWAV201, and the second one (NCT06650319), started in September 2024, evaluating safety and efficacy of LY-M003, an AAV8-gene therapy product.

## Conclusion

While genetic therapies for movement disorders are rapidly evolving with exciting examples of licensed products and life-transformative effects, many hurdles remain. Arguably, the treatment of early-onset, non-neurodegenerative, monogenic disorders with well-understood pathomechanisms, targeting defined brain regions, appear to have had the most tangible benefit to patients, as is evident in AADCD. Longitudinal follow-up studies will be important to understand the longer-term outcome for these patients. Such successes in the field will no doubt help guide future efforts for other similar conditions [94] and also for more challenging complex movement disorders. Better understanding of the pathophysiological processes underpinning Parkinson's Disease will facilitate future development of improved precision therapies that may need combination approaches to address dopamine dysregulation, neuroinflammation, ferroptosis, mitochondrial dysfunction and protein misfolding and aggregation. Adequate targeting of the whole brain is necessary for some movement disorders, but challenging, and may be overcome in time, with improved vectors and better neurosurgical techniques. Further understanding of off-target effects and toxicity in humanized systems (for example, humanized mice models, induced pluripotent stem cell-derived neuronal models) and large animal models will also improve the safety and efficacy of these therapies. Careful development of meaningful clinical outcome measures will facilitate more accurate assessment of efficacy in clinical trials. Understanding the optimal therapeutic window for treatment will also be important; to maximize clinical benefit; it may be important to treat patients either very early in the disease course for neurodevelopmental movement disorders (infancy or even fetal gene therapy) or perhaps before a critical threshold of neuronal loss for neurodegenerative movement disorders. Overall, advancement of such next generation strategies is vital to reduce morbidity and increase life expectancy for patients with movement disorders.
